# Phenotypic and genomic analysis of the emerging poultry pathogen Enterococcus cecorum in UK isolates

**DOI:** 10.1099/mgen.0.001504

**Published:** 2025-09-30

**Authors:** Finn O'Dea, Amandine Dupuis, Joseph Brown, Joseph Edwards, Sammy Kay, Graham P. Stafford, Stephane Mesnage

**Affiliations:** 1 Molecular Microbiology, School of Biosciences, University of Sheffield, Sheffield, UK; 2 Aparon Ltd, Lincoln, UK; 3 School of Clinical Dentistry, 19 Claremont Crescent, University of Sheffield, Sheffield, UK

**Keywords:** antimicrobial resistance, emerging pathogen, *Enterococcus cecorum*, food security, One Health

## Abstract

The rate of *Enterococcus cecorum* infections within poultry (e.g. chickens, racing pigeons and Pekin ducks) has rapidly increased since they were initially reported in the UK and the Netherlands in 2002. *E. cecorum* can infect the free thoracic vertebra of chickens, often leading to carcass rejection at processing plants, incurring large economic costs to farmers. The environmental spread of this pathogen has been facilitated by high levels of antimicrobial resistance, as well as the emergence of divergent clonal lines with a higher pathogenic potential within poultry. Previous genomic studies have explored these characteristics within *E. cecorum* populations in both the USA and France. However, whether *E. cecorum* isolates in other countries show the same genomic traits remains unknown. In the present study, we investigate the properties of 190 *E. cecorum* isolates collected from UK broiler farms between 2021 and 2022. We report the MICs and epidemiological cutoff values for 32 antibiotics and analyse the genome sequence of 39 representative organisms from diseased or healthy broiler farms. We show that UK isolates present phenotypic signs of resistance for 26 antibiotics tested and show that clinical isolates are enriched in a single clade. These strains share properties with pathogenic strains from France and the USA, including conserved cell wall biosynthesis loci, as well as mobile genetic elements involved in the propagation of antibiotic resistance genes. Our results support the idea of a cross-continental spread of virulent *E. cecorum*.

**Impact Statement**

We provide a phenotypic and genotypic analysis of UK *Enterococcus cecorum* isolates showing they group into clades alongside isolates from both the USA and France. This paper gives further evidence to the idea of a transcontinental spread of certain *E. cecorum* lineages which display a higher pathogenic potential within poultry.

## Data Availability

All strains described in this study are available upon request to S. Mesnage. Genome sequences have been uploaded to the BioProject PRJNA1266883 under the BioSample numbers SAMN48512760 to SAMN48512793 and SAMN48894586 (AP_17) (Table S1).

## Introduction

*Enterococcus cecorum* was first reported as *Streptococcus cecorum* in 1983 [1]. It was originally considered a commensal of the intestinal microbiome of healthy chickens, but this organism has since been found to infect a range of poultry species, including chickens, racing pigeons and Pekin ducks [2,4]. Rare cases of septicaemia have also been reported in humans, where domestic animals or contaminated meat are thought to act as possible routes of transmission [5,7]. Outbreaks within flocks often result in frequent carcass rejections by processing plants at large economic costs to farmers [8]. Initial reports of *E. cecorum* infections within poultry were noted within the UK in 2002 [9,10]. Since then, the rate of infection has drastically increased, with the bacteria now being responsible for 7% of total French avian infections (up from 0.3% in 2003) [11]. However, the extent to which this is true in the UK remains unclear as surveillance of outbreaks lags behind other countries . These bacteria colonize the free thoracic vertebra of the bird and can cause spondylitis, osteomyelitis and femoral head necrosis or what is colloquially referred to as kinky back [12,14]. Infections are commonly seen in broiler farms after 28 days [15,16], although detection at earlier stages has been reported [13].

Two major factors contributed to the spread of *E. cecorum*: its high levels of antimicrobial resistance (AMR) [17] and the emergence of divergent clonal lines causing the disease [18,19]. Divergent clonal lines also suggest that this shift in lifestyle from commensal to pathogen has been coupled with large genomic restructuring both in size and content [18,20]. These alterations have resulted in increased virulence, allowing pathogenic strains to outcompete their commensal counterparts and contributing to their spread [21,22].

Previous work has investigated the genetic differences of non-clinical (commensal) and clinical (pathogenic) isolates from both France [23] and America [18]. The trade of poultry between the UK and France means an investigation of UK *E. cecorum* populations is crucial for determining the extent to which these pathogenic clonal lines (found in French broiler farms) have spread internationally. The present study aimed to investigate whether the genetic alterations observed in French/American isolates are conserved in UK *E. cecorum* strains, as well as to determine some of the features which may underpin their pathogenesis. The MICs for 190 UK *E. cecorum* isolates were determined against 32 antibiotics. Isolates with and without phenotypically detectable levels of resistance to a given antibiotic were identified by calculating epidemiological cutoff (ECOFF) values using the ECOFF finder method from the European Committee on Antimicrobial Susceptibility Testing (EUCAST) website. Additionally, 35 isolates from distinct sampling sources and isolation sites were sent for whole-genome sequencing. Genomes were subsequently grouped with respect to previously described clades [23]. Comparative genomic analysis was carried out on non-clinical and clinical isolates (CIs) focussing on AMR/cell wall biosynthesis genes and mobile genetic elements (MGEs) to identify genetic differences which may contribute to the given lifestyle of the organisms.

## Methods

### *E. cecorum* isolation

*E. cecorum* strains were isolated from poultry samples collected from UK poultry farms between 2021 and 2022 (Table S2, available in the online Supplementary Material). No animal was culled to isolate *E. cecorum*. Animals euthanized by local farmers were provided as by-products of their usual practice. The breasts, caeca, femoral head, heart (and heart blood), hock, knee, meat rinsate, pelvis, pericardium, spleen and wings of both infected and healthy birds were swabbed, along with a range of environmental sources not from animal necropsy. Cultures were grown on bile aesculin agar to select for enterococci. Strain identifications were carried out using a MALDI Biotyper sirius IVD System (Bruker) according to the manufacturer’s instructions.

### Bacterial growth conditions

Bacteria in this study were grown on THY broth (per litre; 30 g Todd–Hewitt broth, 10 g yeast extract) or THY agar plates (THY+10 g l^−1^ agar). All isolates were grown at 37℃ in the presence of 5% CO_2_.

### Determining MICs/ECOFF values

MICs of 32 antibiotics (Table S3) were determined using the broth dilution method with a UriDot multipoint replicator. Tenfold serial dilutions of all 190 strains were spotted (1 µl per spot, starting at 10^6^ c.f.u.) on agar containing increasing concentrations of antibiotics. MICs were identified as the concentration of antibiotic which completely inhibited the growth of the bacteria. ECOFF values were calculated based on the distribution of MICs in the population using the method described by EUCAST (EUCAST: MIC and zone distributions and ECOFFs).

### Strain sequencing

Strains for sequencing were grown as overnight cultures to an OD_600_ of >1.5. Samples were spun down at 6,000 r.p.m. for 10 min, and 50 mg of the resulting cell pellet was sent for sequencing. Sequencing was completed by MicrobesNG and Plasmidsaurus using Illumina (MicrobesNG) and Nanopore (Plasmidsaurus) sequencing technology. Genomes were assembled using the pipelines described on these company websites.

### Determining pangenome

Genomes were uploaded to KBase [24] as GenBank files and converted to a genome set using GenomeSet – v1.7.6. This genome set was then input into both mOTU.Pangenome and mOTUpan – v0.3.2 to identify the pangenome [25]. Output files were analysed to determine genes which were core, accessory or unique.

### Phylogenetic analysis

Genomes were uploaded to the Type Strain Genome Server [26] as FASTA files. The Newick output file was uploaded to the Interactive Tree of Life [27] to construct a phylogenetic tree. Loci enriched in clade E organisms were extracted from reference genomes. Genomes were uploaded to Proksee [28] as GenBank files and searched for enriched loci using the blast+ feature. Loci identified were extracted from genomes using SnapGene software and compared with the reference loci using the Clinker sequence alignment tool [29].

### Bioinformatics

#### Identification of mannitol metabolism genes

The mannitol metabolism gene cluster was extracted from a reference genome using SnapGene software. The blast+ feature on Proksee was used to search for the gene cluster in sequenced isolates. SnapGene software was used to extract the gene clusters displaying high sequence homology to that of the reference gene cluster for further comparison using the Clinker sequence alignment tool.

#### Enterococcal polysaccharide antigen cluster analysis

*E. cecorum* genomes were uploaded as FASTA files to Galaxy [30] and annotated with the *Enterococcus faecalis* strain OG1RF genome using the Prokka annotation tool. Annotated genomes were searched for the presence of OG1RF EPA genes. EPA loci identified during this search were extracted from the genomes using SnapGene software and compared to each other using the Clinker sequence alignment tool.

#### Identification of antimicrobial resistance genes and MGEs

Antimicrobial resistance genes (ARGs) were identified using the Comprehensive Antimicrobial Resistance Database (CARD) v.3.2.4 [31] on Proksee and ResFinder [32]. Genomes were uploaded as GenBank files (CARD) or FASTA files (ResFinder) and searched for the presence of ARGs. mobileOG-db [33] on Proksee was used to identify MGEs. Genomes were uploaded as GenBank files, and the mobileOG-db tool was used. Outputs were downloaded as Excel files for further analysis. Tn916 MGEs were identified by searching the reference ID of MGEs identified from the mobileOG-db on UniProt [34]. A blast+ search was used to observe where these elements were inserted into the genome. This was then compared with the insertion site of ARGs. Prophages were identified using a mixture of the Phigaro [35] tool on Proksee along with PHASTEST [36]. Genomes were uploaded as either GenBank (Porksee) or FASTA (PHASTEST) files. Prophage regions identified were downloaded as FASTA files for further analysis. Modules present within these prophages were identified using the PHASTEST output files.

#### Capsule locus analysis

Capsule loci were identified using blast+ on PROKSEE. Loci identified were extracted from the genomes and compared to *Staphylococcus aureus* and *E. cecorum* capsule loci [18] using the Clinker comparison tool. Individual genes were extracted, and protein structures were predicted using AlphaFold 3.0 [37]. PDB structures were then uploaded to Foldseek [38] to find structural homologues.

#### Identification of CRISPR/phage defence systems

CRISPR systems were identified using the CRISPR/Cas [39] finder tool on Proksee. Genomes were uploaded as GenBank files, and the genomic location/type of CRISPR system present in the isolate was noted. Phage defence systems were identified using DefenceFinder [39,41]. Genomes were uploaded as FASTA files and searched for any defence systems present. Output files were downloaded for further analysis.

## Results and discussion

### Analysis of 190 UK *E. cecorum* isolates reveals high levels of antibiotic resistance

MICs for 32 antibiotics [chosen based on clinical usage in humans/poultry (Table S3)] were determined against all 190 * E. cecorum* strains in our collection (Table 1) and Fig. S1). ECOFF95 values were determined for all antibiotics except fosfomycin, spectinomycin and cotrimoxazole, which displayed very high MICs. The ECOFF95 values revealed phenotypic signs of resistance for 26 out of 29 antibiotics. The highest levels of resistance observed were against the tetracycline class of antibiotics, with 54% of isolates being resistant to tetracycline and doxycycline. Additionally, high levels of resistance were observed against *β*-lactams [cefotaxime (17%), phenoxymethylpenicillin (12%)], as well as a range of miscellaneous antibiotics [tiamulin (22%)/nitrofurantoin (8%)]. Multidrug resistance was seen across the population with one strain (AP_95) being resistant to 12 antibiotics from five different classes. Of particular concern was the observed resistance to antibiotics commonly used to treat *E. cecorum* infections within the UK, especially within CIs. These include enrofloxacin (9 strains, all CIs), amoxicillin (7 strains, 86% CIs), phenoxymethylpenicillin (23 strains, 83% CIs) and lincomycin/spectinomycin (34 isolates, 5% CIs), as well as doxycycline (102 strains, 88% CIs). Enrofloxacin resistance within CIs is likely to result from the usage of this antibiotic to treat *E. coli* infections [42]. Resistance is therefore likely to increase the risk of *E. cecorum* infections following dysbiosis caused by this antibiotic treatment.

**Table 1.** ECOFF and MICs of antibiotics for *E. cecorum* isolates

*Concentrations are given in micrograms per millilitre. The black vertical lines correspond to ECOFF values determined in this study; when available, ECOFF values determined in a recent study characterizing French isolates are indicated with red lines [17]; grey vertical lines indicate that ECOFF values were identical in both studies. Antibiotics with ECOFF values >256 µg ml−1 are highlighted in grey.

Resistance profiles were similar between UK and French broiler farm isolates. There were, however, some clear differences in a number of antibiotics tested. Amoxicillin MICs observed in UK isolates were higher than French isolates with an MIC_90_ of 0.125 µg ml^−1^ in France compared to 2 µg ml^−1^ in the UK. UK isolates also seemed to display higher levels of resistance to daptomycin and gentamicin but lower levels of resistance to glycopeptides such as vancomycin and teicoplanin.

### Phylogenetic analysis of UK *E. cecorum* isolates reveals clustering of clinical strains and a novel subclade

**Fig. 1.**
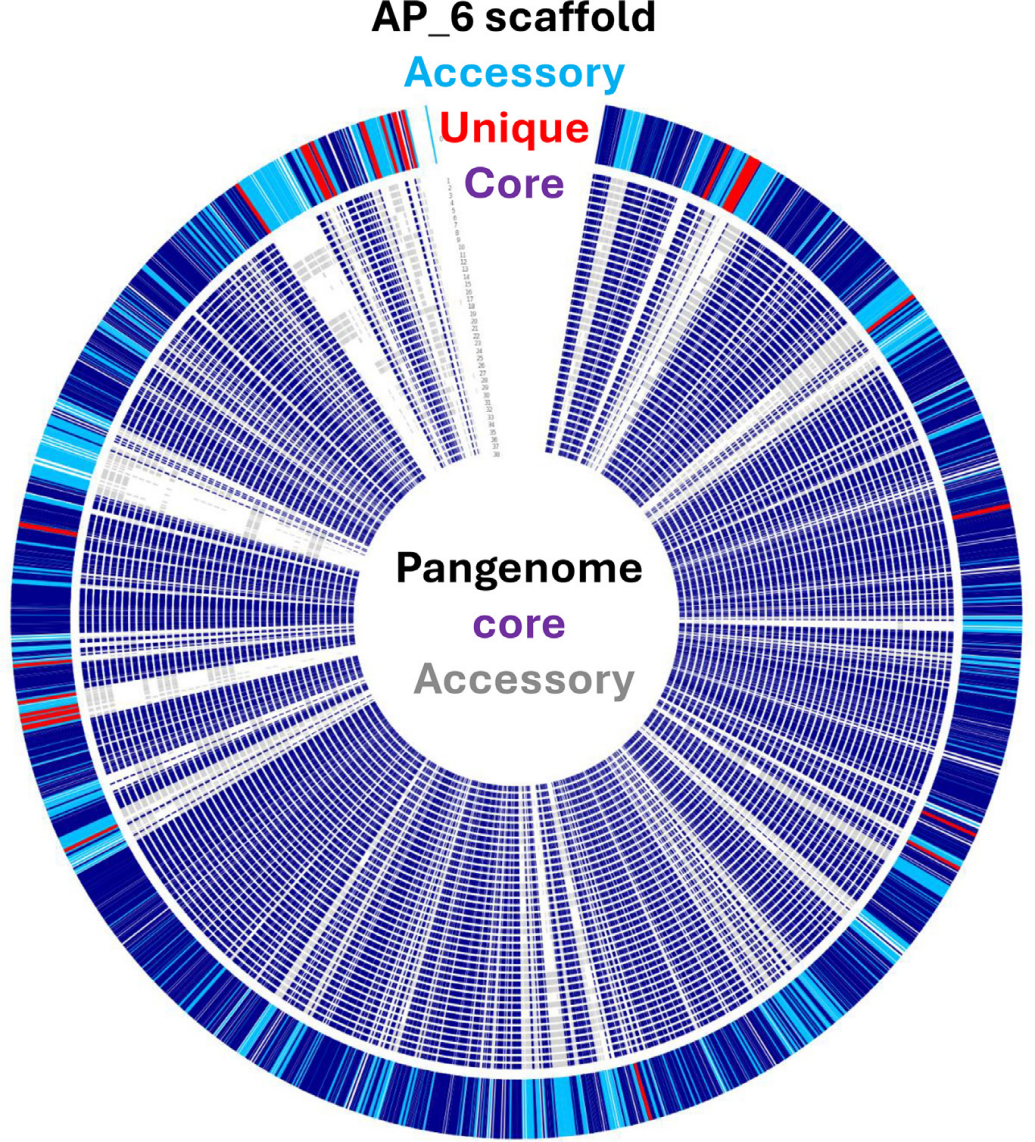
Pangenome of 39 UK *E. cecorum* isolates. Pangenome of UK *E. cecorum* isolates with core genes shown in purple and non-core genes shown in grey.

Representative strains from various isolation sources and locations were chosen for sequencing. In total, whole-genome sequencing was performed on 19 CIs (from diseased birds), 7 non-CIs (from non-diseased birds) and 9 isolates from the environment collected in the UK between March 2021 and April 2022 (Table S1). A sequenced type strain (NCTC12421) and three collection strains (DSMZ11364, DSMZ109010 and DSMZ100908) were also included for comparison. Sequencing was performed using Illumina and Nanopore sequencing technology with an average coverage of 139.7× and an N_50_ between 74 and 208 kbp. The average genome size of isolates was ~2.35 Mbp with the smallest being 1.97 Mbp and the largest being 2.9 Mbp. Smaller genome sizes were observed in CIs with an average genome size of 2.2 Mbp compared to 2.6 Mbp in non-CIs. This smaller genome size highlights the genomic restructuring seen previously in CIs [18,23]. A total of 90,862 CDSs were found, averaging 2,330 CDSs per genome across the 39 strains. From this, a pangenome containing 7,268 gene clusters was identified. The pangenome (Fig. 1) was broken down into a core genome containing 1,384 genes, an accessory genome containing 4,335 genes and a unique genome of 1,594 genes. This core genome size mirrors what has been observed elsewhere [23].

**Fig. 2.**
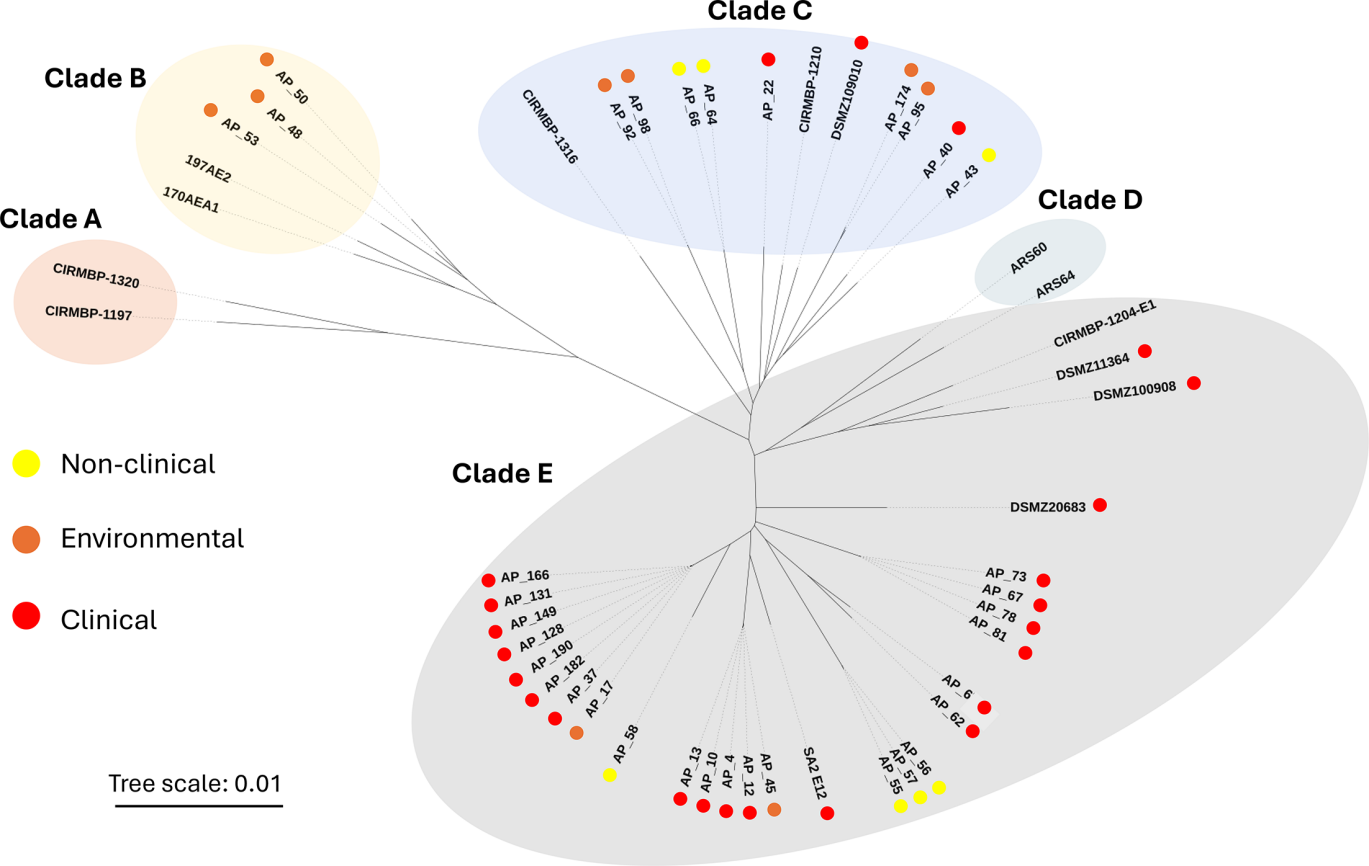
Phylogenetic tree of 39 *E. cecorum* isolates with clinical status and clade organization displayed. The phylogenetic tree of sequenced strains shows grouping into four clades: clade B (orange), clade C (blue), clade D (teal) and clade E (black). Red (clinical), yellow (non-clinical) and orange (environmental) dots indicate the clinical status of bird’s isolates taken from. Unlabelled nodes correspond to reference strains from [23]. Scale bar refers to the relative measure of evolutionary distance.

Phylogenetic relationships between isolates were subsequently investigated, revealing strains grouped into three out of five previously characterized clades [23] (Fig. 2). Most isolates [66.7% (26 out of 39)] clustered in clade E. In contrast, 7.6% (3 out of 39) of isolates clustered into clade B, and 25.6% (10 out of 39) clustered into clade C (Fig. 2). No clade A or D isolates were found in this study. This is unsurprising for clade A, as organisms belonging to this clade were isolated from humans who were not sampled here. It is, however, surprising that no clade D organisms were identified. Amongst clade E organisms, 76.9% (20 out of 26) were CIs, 7.7% (2 out of 26) were environmental isolates and 15.4% (4 out of 26) were non-CIs. In contrast, clade B contained no CIs, and only 30% (3 out of 10) of clade C isolates were clinical. This suggested that pathogenic, clinical strains isolated in the UK are phylogenetically related and mostly found in clade E. The observation also reinforces the idea that *E. cecorum* clinical strains are becoming increasingly phylogenetically distant from non-CIs. These alterations to the genome are likely to contribute to their ability to infect birds with a greater efficiency and to persist in poultry for longer periods of time.

To further investigate the diversity within the UK clade E isolates and compare them to strains previously described, we grouped isolates into subclades. One representative strain of each of the 13 subclades defined with French isolates was added to the 39 UK isolates, and a phylogenetic tree was constructed (Fig. S2). Grouping of these organisms revealed the presence of a divergent subclade (called E14) made up of eight isolates. The emergence of this new subclade may suggest a divergence in UK *E. cecorum* isolates away from what is seen elsewhere in continental Europe and America.

### UK isolates lack locus associated with commensal isolates

Mannitol metabolic genes have previously been described as reporter genes for non-clinical strains due to their absence in pathogenic isolates [18]. These genes include the mannitol operon activator, Blg family protein, PTS system mannitol-specific IIa component and the mannitol-1-phosphate 5-dehydrogenase. In contrast to what has been observed elsewhere, this gene cluster was present in a single CI (AP_40). Additionally, only 1 out of the 8 non-clinical isolates and 4 out of the 39 total isolates contained this cluster (Table S4). These strains did, however, contain proteins displaying low sequence homology (<29%) to those described above. Together, these differences in mannitol metabolism and the emergence of an additional subclade within clade E suggest that UK isolates may be diverging away from those found in continental Europe, possibly to better suit conditions in UK broiler farms.

### Antibiotic resistance genes

**Fig. 3.**
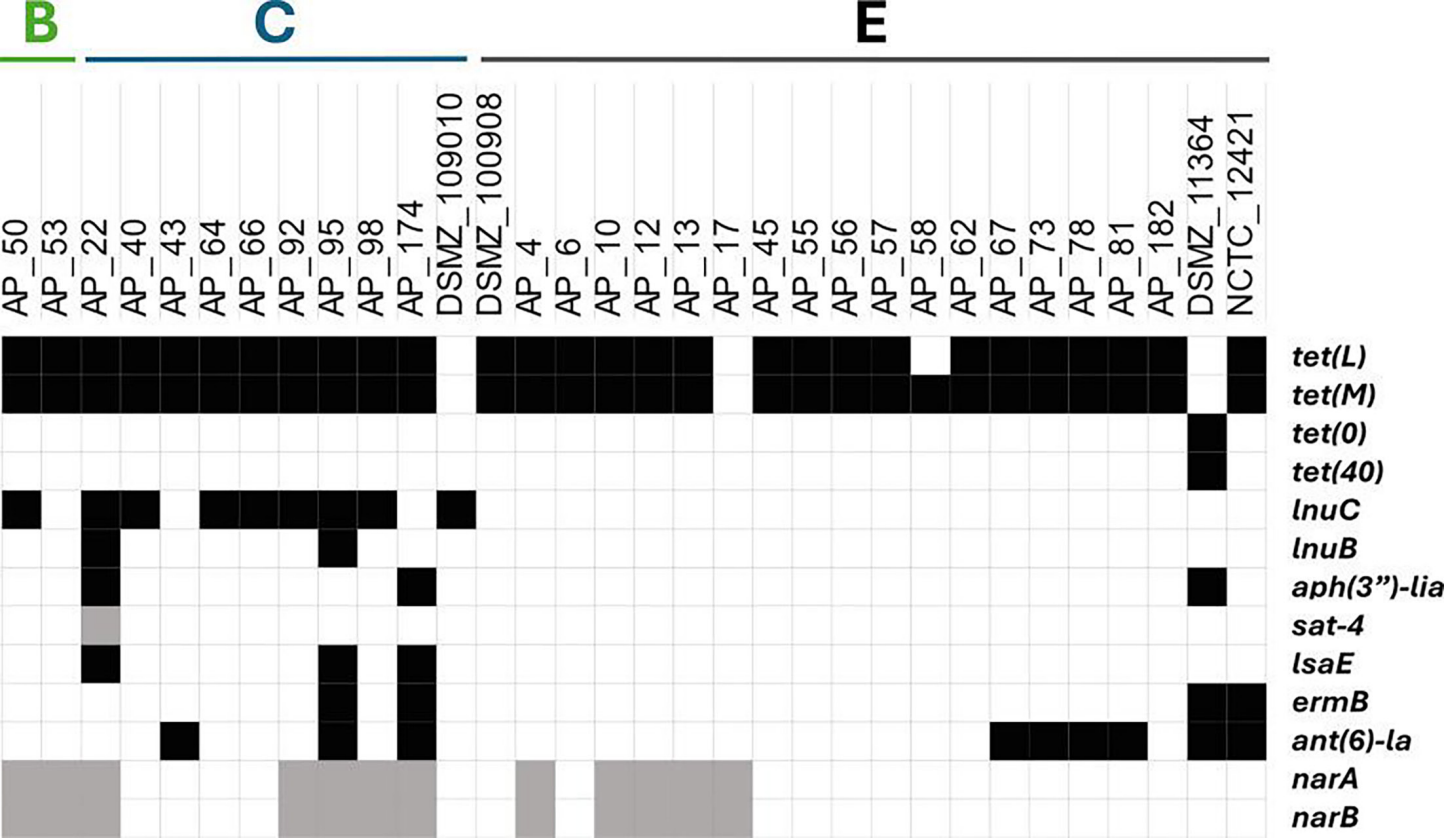
ARGs present in sequenced strains. ARGs present in sequenced strains showed high levels of resistance to the tetracycline and lincosamide class antibiotics. Clades of organisms are shown above. Black squares indicate where ARGs match phenotypic signs of resistance [narasin (narA/narB) and streptothricin (sat-4) not tested].

To compare genotype to phenotype in terms of antibiotic resistance, we searched the 39 sequenced genomes for the presence of ARGs using CARD and ResFinder (Fig. 3). A total of 84.6% (33 out of 39) of genomes contained at least 1 ARG, and a total of 13 different ARGs were identified. Of these, 67% (22 out of 33) contained ARGs against different antibiotic classes, with 14 strains containing ARGs against 2 antibiotics, 4 strains against 3, 2 strains against 4 and 3 strains against more than 4. The ARGs present closely mirrored the phenotypic resistance profiles, with the *tet(L)/tet(M*) ARGs being the most observed within sequenced isolates [present in 64.1% (25 out of 39) and 76.9% (30 out of 39) of strains, respectively]. The *tet(O)/tet(40*) resistance genes were also identified; however, this was only present in a single isolate. Other ARGs were also observed with 23.1% of isolates containing *ant(6)-la* (resistance to aminoglycoside antibiotics), 15.4% containing *ermB* (resistance to MLS antibiotics)*,* 30.8% containing *NarA/NarB* (resistance to narasin) and 20.5% possessing *lnuC* (resistance to lincosamide antibiotics). Resistance profiles did slightly differ between clade types, with 87.5% of *lnuC* (resistance to lincomycin) containing isolates occurring in clade B and 50% of *ermB* and 66.7% of *ant(6)-la* containing isolates being found in clade E. Clades B and C contained the most ARGs on average, with four and five ARGs per strain, respectively, compared to two for clade E. Phenotypic signs of resistance mirrored ARGs present, in agreement with what has been observed elsewhere [20,23] with the general trend of non-CIs usually possessing more ARGs than their clinical counterparts, as well as the common observation of ARGs such as *tet(L)/tet(M)*, *ant(6)-la* and *lnuC*.

To further investigate the genetic mechanisms which underpin the phenotypic signs of resistance observed for commonly used poultry antibiotics where ARGs were not identified (amoxicillin and enrofloxacin), we looked for point mutations within the genes which these antibiotics target. Point mutations conferring resistance to amoxicillin and cotrimoxazole, previously described as PBP2x, were identified in isolates displaying phenotypic signs of resistance (Met346Thr, Trp377Ser and Leu382Ile in strains AP_66 and AP_98) and could therefore account for resistance to these antibiotics in *E. cecorum*. Mutations in a GyrA residue previously associated with resistance to enrofloxacin were also detected in strains AP_92 and AP_98 (Ser83Val) [20].

Interestingly, several strains which displayed phenotypic signs of resistance to a given antibiotic lacked related ARGs or expected mutations in the genes encoding antibiotic targets. These were true for resistance to streptomycin in AP_64, AP_66, AP_131, AP_149, AP_92 and AP_190; erythromycin in AP_55, AP_56 and AP_57; and lincomycin in AP_55, AP_56, AP_57 and AP_81, potentially suggesting some novel form of resistance in these isolates. The ARGs and point mutations present within isolates mirror what is seen in the phenotypic resistance profiles with high levels of tetracycline (*tet*genes), tiamulin (*IsaE*) and lincomycin (*lnuC/lnuB*) being observed. Additionally, ARGs and point mutations conferring resistance to antibiotics used in the treatment of *E. cecorum* infections (in poultry) were also observed, including doxycycline (*tet* genes), amoxicillin/phenoxymethylpenicillin (mutations in PBP2x), lincomycin/spectinomycin (*lnuC/lnuB*) and enrofloxacin (mutations in GyrA). Combining determination of ECOFF values and genomic analyses provides insights into resistance mechanisms and useful information to inform therapeutic strategies to eradicate *E. cecorum* infections moving forward.

### Analysis of cell envelope biosynthesis genes

**Fig. 4.**
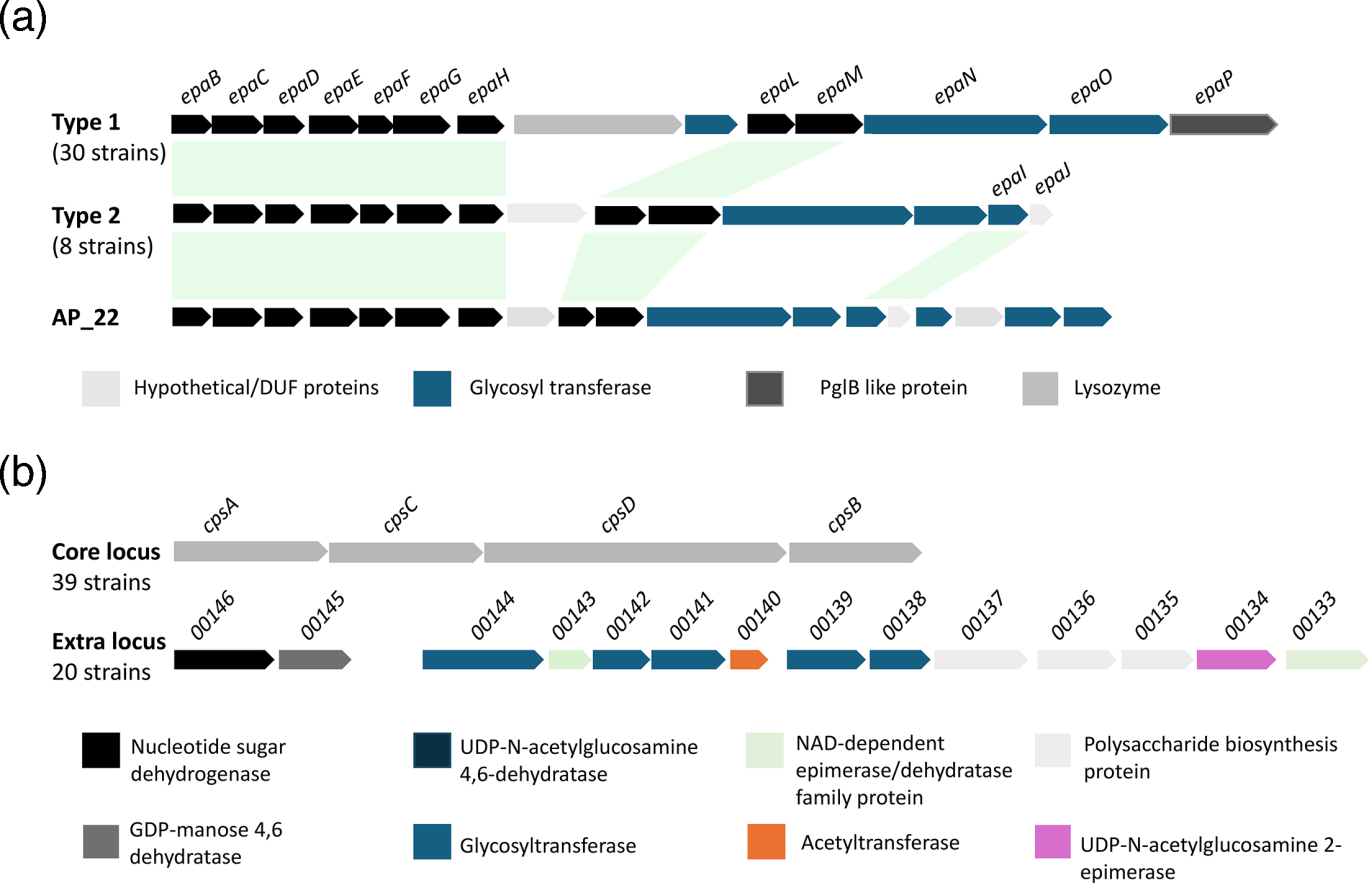
*epa* and capsule loci in *E. cecorum*. (**a**) Divergent *epa* loci exist in *E. cecorum*; pathogenic strains mostly contain a type 1 cluster, whilst non-clinical strains display a type 2 cluster. A variant of the type 2 locus was identified in AP_22. (**b**) The four-gene core capsule locus is found in all strains, whilst the additional capsule locus is only found in a proportion of clade E isolates.

EPA is a key cell envelope component present in a range of clinically important enterococci [43]. This polysaccharide plays a crucial role in a variety of processes including immune evasion, colonization of a niche and phage infection [44,48], making it an important cell surface structure to investigate. Bioinformatic analysis of sequenced genomes revealed the presence of two distinct types of EPA loci, classified here as type 1 and type 2. Type 1 organisms contain a 14-gene operon (*epaB-H* and *epaL-M*, with two additional genes encoding a glycosyltransferase and glycosylhydrolase between *epaH *and *L*), whilst type 2 organisms contain a 13-gene operon (*epaB-H*, *L*, *M*, *I* and *J* with two additional glycosyltransferases between *epaM* and *I*) (Fig. 4a). A third type of EPA cluster (type 3) has previously been described in *E. cecorum* which contains only 7 genes (*epaB-H*); however, no organisms containing this locus type were found in our study. A slightly divergent EPA locus was identified within one clinical strain (AP_22). This locus contains 5 glycosyltransferases which differ from those seen in other type 2 organisms, as well as an additional gene encoding a protein of unknown function.

The type 1 EPA locus was the most abundant with 76.9% (30 out of 39) of strains possessing this operon. In contrast, type 2 systems were only found in 23.1% (9 out of 39) of strains. Additionally, the type 1 locus was highly enriched in CIs, with 91% (21 out of 23) of CIs containing this locus type. Contrast that with environmental and non-CIs, where only 66.7% (6 out of 9) and 37.5% (3 out of 8) contain the type 1 locus, respectively. Unsurprisingly, due to its prevalence in CIs, type 1 systems are also highly enriched in clade E, with 92.3% of isolates containing this locus in contrast to 33.3% of clade B and 50% of clade C isolates.

*E. cecorum* possesses two capsular polysaccharide-encoding loci. The first is a four-gene core operon (Fig. 4b) which shows similarity to the *Enterococcus faecium* capsule locus and was present in all the sequenced isolates. An additional polysaccharide-encoding locus is found within *E. cecorum* strains (Fig. 4b). This locus is located upstream of the core *E. cecorum* capsule operon and contains a variety of genes potentially involved in the production of a capsular polysaccharide. Genes present in this locus show both sequence and putative structural homology to capsule genes present in the *S. aureus* capsule locus including homologues to *cpsD*, *E*, *F*, *G*, *M* and *N*. This operon was highly enriched in clade E [present in 92.3% (24 out of 26) of clade E isolates], yet only present in 20% (2 out of 10) of clade C isolates and absent in clade B. Divergence in this additional polysaccharide-encoding locus is observed within clade E, particularly in non-CIs (Fig. S3).

Conservation of the type 1 EPA locus along with the presence of the secondary capsular polysaccharide locus suggests an alteration to the cell envelope composition of CIs which may contribute to their virulence. This cell envelope remodelling has been observed in other enterococcal species. For example, *E. faecalis* strains are grouped into serotypes based on the capsular polysaccharide operons they contain, with serotypes A and B encompassing non-clinical/non-capsule-producing isolates and serotypes C and D encompassing clinical/capsule-producing isolates [49]. This may reflect what is happening in *E. cecorum* where clinical strains possess a secondary capsule locus for the production of a functional capsule, in turn increasing their virulence. The addition of EPA genes within clinical strains may also suggest the production of a more complex polysaccharide which may again increase virulence. The divergent EPA locus found in AP_22 suggests that alterations to the EPA locus are ongoing, potentially to adapt to the newfound niche of CIs.

### Mobile genetic elements

Horizontal gene transfer is an important process which drives bacterial evolution. The process confers numerous advantages to the host, improving both fitness and virulence. MGEs encode the machinery needed for this gene transfer, making them important components to investigate. To do this, the mobileOG database was utilized to identify all MGEs present in sequenced isolates. MGEs are grouped into four categories: integration/excision, replication/recombination, stability/defence and transfer. Clade B isolates contained the most MGEs with 263 per strain, followed by clade C (184) and finally E (142). Despite possessing the highest number of MGEs related to integration/excision (167) and stability/defence (31), clade B organisms contained the lowest number related to replication/recombination/repair (50) and transfer (15). Clade C (58) organisms were the most enriched in replication/recombination/repair and transfer MGEs, respectively. The integration/excision class was the most commonly found MGEs in three of the four clades (B, C and E).

Prophages are MGEs that actively contribute to shaping bacterial evolution [50,51]. They contribute to the horizontal transfer of bacterial genes contributing to fitness, virulence and AMR. To identify and characterize prophage within * E. cecorum* isolates, genomes were searched using both Phigaro and PHASTEST. Nineteen complete and three incomplete prophages were identified in ten strains at distinct genomic locations (Table 2). Prophages varied in size (largest=57,956 bp, smallest=4,373 bp) and gene content. Clade C organisms contain the highest amount of complete prophage, followed by clade E organisms (an average of one prophage per genome compared to three). Prophage was further investigated to look for differences in gene content by searching for the presence of six modules needed for the production of a functional phage (Table S5) (lysogeny, replication, transcriptional regulation, head and tail morphogenesis, DNA packaging and lysis). Out of the prophages present, NC_048631 belonging to strain NCTC_12421 was the most complete prophage, containing all modules required for excision from the bacterial genome.

**Table 2.** Prophages identified within *E. cecorum* isolates

Isolate name	Length (bp)	GC%	Completeness*	Closest phage (NCBI)	Accession no.
**AP_22**	51,227	37.03	Intact	Lister_LP_101	NC_024387
**AP_50**	20,566	37.29	Questionable	Escher_SH2026Stx1	NC_049919
**AP_56**	26,898	45.34	Intact	Strept_EJ_1	NC_005294
	38,143	35.98	Intact	Strept_A25	NC_028697
**AP_57**	26,898	45.34	Intact	Strept_EJ_1	NC_005294
	38,143	35.98	Intact	Strept_A25	NC_028697
**AP_64**	47,005	37.51	Intact	Entero_EFC_1	NC_025453
	43,487	26.22	Intact	Strept_A25	NC_028697
	28,966	38.51	Questionable	Staphy_SPbeta_like	NC_029119
**AP_66**	40,064	37.61	Intact	Entero_EFC_1	NC_025453
	47,071	36.08	Intact	Strept_A25	NC_028697
	4,373	34.22	Questionable	Staphy_SPbeta_like	NC_029119
**AP_67**	36,241	41.12	Intact	Strept_EJ_1	NC_005294
**AP_73**	40,840	41.42	Intact	Strept_EJ_1	NC_005294
**AP_92**	40,460	37.31	Intact	Lactoc_bIL285	NC_002666
	47,224	36.96	Intact	Lister_LP_101	NC_024387
	39,685	35.32	Intact	Lactoc_50101	NC_031040
**AP_98**	38,366	37.32	Intact	Lactoc_bIL285	NC_002666
	47,224	36.96	Intact	Lister_B025	NC_009812
	37,520	35.54	Intact	Lactoc_50101	NC_031040
**NCTC_12421**	57,956	40.41	Intact	Bacill_phi105	NC_048631
	32,575	36.50	Intact	Lactoc_50101	NC_031040

* Based on PHASTEST prediction software.

To see how these prophages may be contributing to bacterial fitness, prophage regions were searched for the presence of genes not involved in the phage lifecycle. Multiple genes were identified linked to a variety of processes which may contribute to improving overall fitness. One gene commonly observed encoded a type II toxin–antitoxin system (TAs; HicB family antitoxin) involved in the protection of host cells from extracellular toxins. These were found within the prophage boundaries of AP_64_P2, AP_66_P2, AP_92_P2 and AP_98_P2. Importer genes involved in the uptake of multiple substrates such as phosphate, sugars and nucleotides were also identified within prophages AP_22, AP_50_P1 and AP_66_P2. Finally, the *Streptomyces* ARG *ant(1)-Ia* (associated with resistance to aminoglycosides) along with the bacitracin transport permease BcrB (associated with resistance to bacitracin) was found within DSMZ20683_P1, highlighting the role of phage in the propagation of resistance genes.

Next, the CRISPR systems present within isolates were investigated. These systems are known to shape the mobilome by preventing/enabling the uptake of foreign genetic material. CRISPR systems carry out different roles and can often be used as markers for virulence [18]. Three CRISPR systems were identified in isolates including type-IIa, -IIIa and -IC systems. Clade E isolates contained each type of CRISPR system, with four isolates each containing type-IIa and type-IIIa systems, and one isolate containing a type-IC system. Clade C only contained type-IIa (one isolate) and -IIIa (two isolates) systems, whereas only a type-IIa system was observed in clade B (one isolate). Type-IIIa CRISPR systems, to the best of our knowledge, have not previously been described in *E. cecorum* or any other enterococci [52]. It was also observed that out of the 11 isolates that contained prophage, only 3 had functional CRISPR systems. This is unsurprising as these systems are known to play a role in preventing phage infection [53]. Additionally, links between CRISPR systems and ARGs present within genomes were observed, with 78% of *lnuC*-containing isolates and 67% of *aph(3’)-ila*-containing isolates lacking a functional CRISPR system. This again suggests that CRISPR systems may be working to prevent the uptake of foreign genetic material, in turn, preventing new ARGs from integrating into the genome.

Finally, genomes were searched for the presence of phage defence systems. These systems are known to prevent phage infections but also digest other foreign genetic material such as plasmids [54]. Five main classes of phage defence systems were found in our study (classes I, II, III, IV and VI). These systems differ in their defensive mechanisms, with class I systems incorporating restriction modification systems (RMs), class II incorporating the aforementioned CRISPR systems, class III incorporating abortive infection systems (AIs), class IV incorporating TAs and, finally, class VI incorporating CBASS (Cyclic-oligonucleotide-Based Anti-phage Signalling System; only found in DSMZ100908). A variety of other systems were also identified which could not be grouped into these five classes. On average, clades C and E contained the lowest number of phage defence systems with an average of 2.3 and 2.8 systems per isolate. In contrast, clade B contained 5.3 systems per isolate. The distribution of these systems also differed across clades. Class I defence systems were predominantly found in clade B with isolates containing an average of 3.7 RM systems (including RM type-I, -II, -IIG, -III and -IV); in contrast, clades C and E contained only 1.5 and 0.5 RMs, respectively. Class III systems were more commonly found in clade E with an average of 1.1 AIs per isolate compared to 0.7 for clades B and 0.6 for clade C. This observation may, however, be due to the larger average genome sizes in clades B. Unsurprisingly, it was observed that isolates containing prophage had a lower-than-average number of defence systems for their clade (clade E 2/2.3, clade B 2/5.3 and clade C 2.5/2.8), suggesting that these isolates have an increased susceptibility to phage infections.

Altogether, a variety of MGEs and other factors influencing the mobilome were identified within isolates. In most cases, these MGEs conferred improved bacterial fitness via the acquisition of resistance genes, nutrient transporters and anti-toxin systems. An average of 0.6 prophage was identified per isolate, which is significantly less than is found within other enterococcal species such as *E. faecalis* [55,56] and *E. faecium* [57,58]. CRISPR systems were also common within isolates, with 17 out of 39 sequenced strains containing a functional CRISPR system. CRISPR systems IIa and IC were identified which are commonly found in enterococci. Additionally, the type-IIIa system was found within some isolates. This system has not previously been identified in enterococci and was only observed in clinical/environmental isolates, suggesting a possible role in virulence.

## Concluding remarks

The emergence of *E. cecorum* as a poultry pathogen over the past 20 years has led to increased interest in the genetic makeup of the organism. Studies suggest that this switch in lifestyle has been accompanied by genomic restructuring allowing pathogenic strains to outcompete commensals and spread internationally. The present study characterized ECOFF95 values for 32 antibiotics against 190 UK *E. cecorum* isolates with high levels of resistance observed against tetracycline, doxycycline, daptomycin and gentamicin. This resistance occurred through both acquisition of resistance genes and point mutations within the genes encoding their targets. Of particular concern was the observed resistance to antibiotics commonly used to treat *E. cecorum* infections within poultry, most of which was found within CIs. Whole-genome sequencing of 35 representative isolates revealed strains clustered into three clades with a single clade (E) making up nearly 80% of isolates. Isolates belonging to this clade contained divergent loci encoding the enterococcal polysaccharide antigen and capsular polysaccharides. The capsular polysaccharide in these isolates is encoded by two loci, one core locus and one additional locus which appears only in this clade. This is similar to what is observed within *E. faecalis*. EPA operons found in clade E were mostly divergent from what was observed within other clades, with organisms possessing more complex loci. Additional analysis of this clade also revealed a group of UK isolates clustered into a new subclade which has diverged away from those seen in continental Europe and America. Together, these data give further evidence to the transcontinental spread of certain *E. cecorum* lineages, and the emergence of an additional subclade also suggests that UK isolates may be becoming more phylogenetically distant to isolates from other countries. These clinical strains possess distinct cell envelope biosynthesis genes encoding cell surface structures closely linked to virulence. Observed resistance to antibiotics common in the treatment of *E. cecorum* infections also suggests that a rethink of the *E. cecorum* treatment plan is required.

## Supplementary material

10.1099/mgen.0.001504 Supplementary Material 1.

## Abbreviations

AIs: abortive infection systems
AMR: antimicrobial resistance
ARGs: antimicrobial resistance genes
CARD: Comprehensive Antimicrobial Resistance Database
CIs: clinical isolates
ECOFF: epidemiological cutoff
EUCAST: European Committee on Antimicrobial Susceptibility Testing
MGEs: mobile genetic elements
RMs: restriction modification systems
TAs: toxin–antitoxin system
