# Supplementary material for: Phenotypic and genomic analysis of the emerging poultry pathogen Enterococcus cecorum in UK isolates
Source: Microb Genom. 2025 Sep 30;11(9):001504. doi: 10.1099/mgen.0.001504 (PMC12483430; doi:10.1099/mgen.0.001504)
Supplement: Supplementary Material 1. [file mgen-11-01504-s001.pdf]

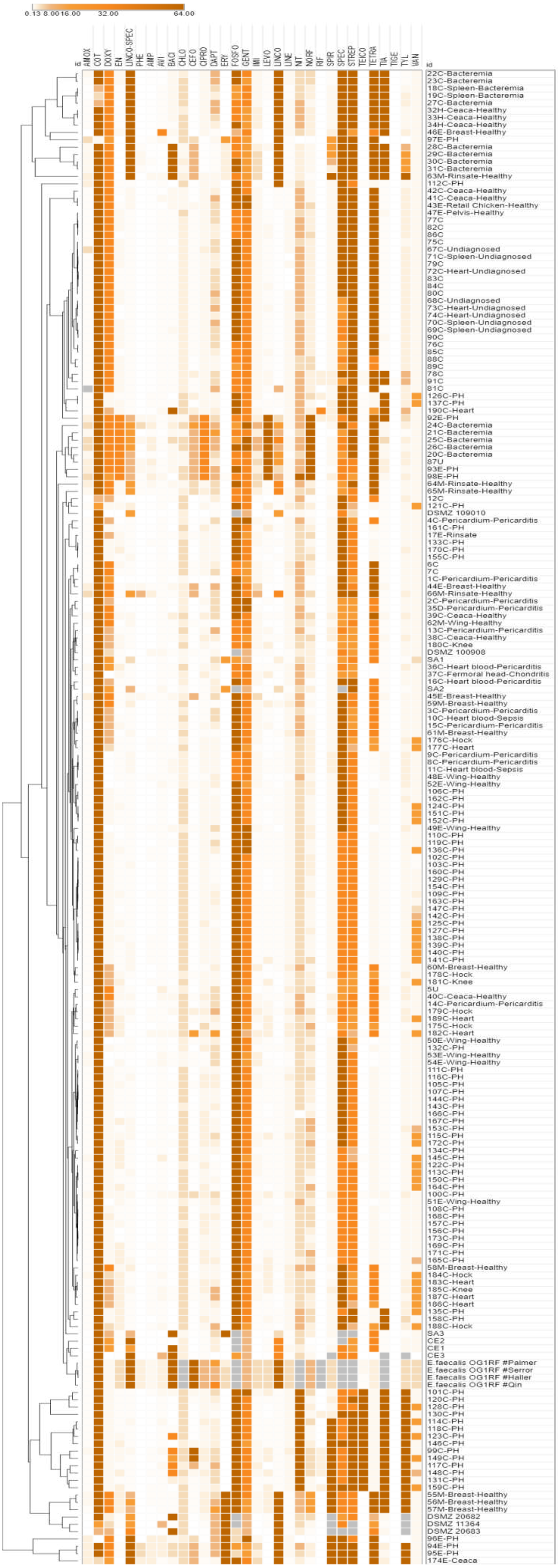

Figure S1. MICs for 32 antibiotics against 190 UK *E. cecorum* isolates. MICs of 32 antibiotics were determined as the minimum concentration of antibiotic required to completely inhibited growth using the broth dilution method with a UriDot multipoint replicator.

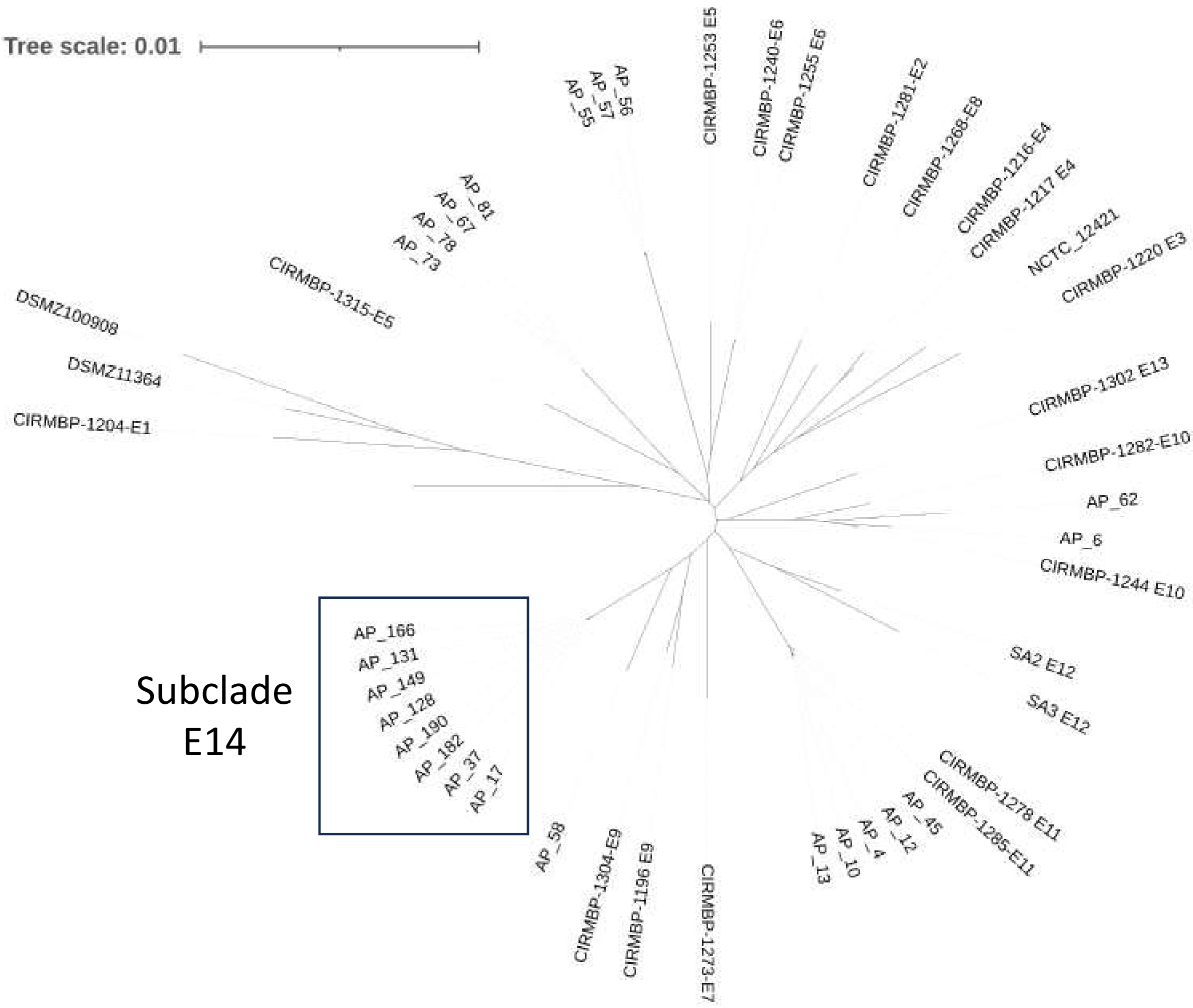

Figure S2. Phylogenetic tree of clade E organisms. Phylogenetic tree shows how UK isolates (AP strains) group with reference strains of each of the 13 subclades found within clade E. This grouping allows the identification of a new subclade here named subclade E14. Scale bar refers to the relative measure of evolutionary distance.

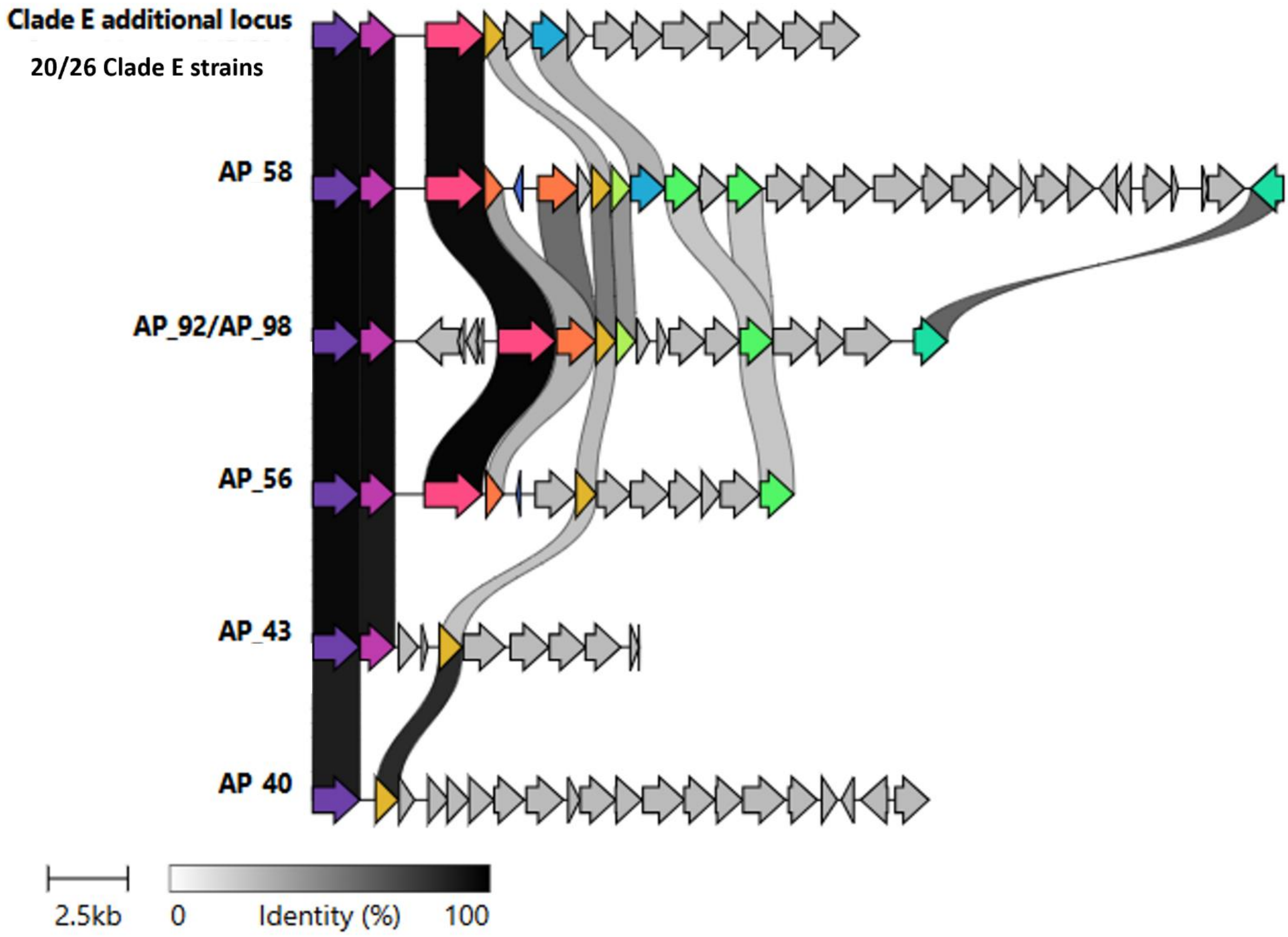

Figure S3. Additional capsular polysaccharide loci found within *E. cecorum* isolates. A secondary capsular polysaccharide biosynthetic locus is found within clade E organisms. Despite most of the organisms possessing identical loci, some divergence does exist within the population.

Table S1. Chosen *E. cecorum* strains for whole genome sequencing.

Bioproject number	Strain number	Status	Source	Clinical History	Age (days)	Collection date
SAMN48512760	**4**	Clinical	Pericardium	Pericarditis	Unkown	03/04/2021
SAMN48512761	**6**	Clinical			Unkown	22/03/2021
SAMN48512762	**10**	Clinical	Heart Blood	Putative sepsis	Unkown	23/03/2021
SAMN48512763	**12**	Clinical			Unkown	24/03/2021
SAMN48512764	**13**	Clinical	Pericardium	Pericarditis	22	25/03/2021
SAMN48894586	**17**	environmental	meat rinsate			23/06/2021
SAMN48512765	**22**	Clinical		Bacteremia	15	26/03/2021
SAMN48512766	**37**	Clinical	Fermoral Head	Fermoral	29	27/03/2021
SAMN48512767	**40**	Clinical	Caeca	Healthy	364	22/07/2021
SAMN48512768	**43**	Environmental	Retail Chicken	Healthy	N/A	30/09/2021
SAMN48512769	**45**	Environmental	Breast	Healthy	N/A	28/06/2021
SAMN48512770	**48**	Environmental	Wing	Healthy	N/A	11/10/2021
SAMN48512771	**50**	Environmental	Wing	Healthy	N/A	12/10/2021
SAMN48512772	**53**	Environmental	Wing	Healthy	N/A	13/10/2021
SAMN48512773	**55**	Commensal	Breast	Healthy	N/A	23/08/2022
SAMN48512774	**56**	Commensal	Breast	Healthy	N/A	15/06/2021
SAMN48512775	**57**	Commensal	Breast	Healthy	N/A	20/08/2022
SAMN48512776	**58**	Commensal	Breast	Healthy	N/A	21/08/2022
SAMN48512777	**62**	Commensal	Wing	Healthy	N/A	15/03/2021
SAMN48512778	**64**	Commensal	Rinsate	Healthy	N/A	21/02/2022
SAMN48512779	**66**	Commensal	Rinsate	Healthy	N/A	22/02/2022
SAMN48512780	**67**	Clinical		Lameness -	23	23/02/2022
SAMN48512781	**73**	Clinical	Heart	Lameness -	23	23/02/2022
SAMN48512782	**78**	Clinical				04/01/2021
SAMN48512783	**81**	Clinical				05/01/2021
SAMN48512784	**92**	Environmental	Processing House			22/04/2022
SAMN48512785	**95**	Environmental	Processing House			23/04/2022
SAMN48512786	**98**	Environmental	Processing House			24/04/2022
SAMN48512787	**128**	Clinical	PHS		15 day	25/04/2022
SAMN48512788	**131**	Clinical	PHS		15 day	26/04/2022
SAMN48512789	**149**	Clinical	PHS		15 day	27/04/2022
SAMN48512790	**166**	Clinical	PHS		15 day	28/04/2022
SAMN48512791	**174**	Environmental	Caeca			23/06/2022
SAMN48512792	**182**	Clinical	Heart			27/05/2022
SAMN48512793	**190**	Clinical	Heart			28/05/2022

Table S2. Description of *E. cecorum* UK isolates used in this study.

Strain	Status	Source	Clinical History	Age (days)	Date
**1**	Clinical	Pericardium	Pericarditis	22	01/03/2021
**2**	Clinical	Pericardium	Pericarditis	22	03/03/2021
**3**	Clinical	Pericardium	Pericarditis	unknow	04/03/2021
**4**	Clinical	Pericardium	Pericarditis	Unkown	03/04/2021
**5**	Healthy	Caeca	Healthy	35	05/03/2021
**6**	Clinical	Unknown	Unknown	unknow	22/03/2021
**7**	Clinical	Unknown	Unknown	unknow	06/03/2021
**8**	Clinical	Pericardium	Pericarditis	unknow	07/03/2021
**9**	Clinical	Pericardium	Pericarditis	unknow	07/03/2021
**10**	Clinical	Heart Blood	Putative sepsis	unknow	23/03/2021
**11**	Clinical	Heart Blood	Putative sepsis	unknow	08/03/2021
**12**	Clinical	Unknown	Unknown	unknow	24/03/2021
**13**	Clinical	Pericardium	Pericarditis	22	25/03/2021
**14**	Clinical	Pericardium	Pericarditis	22	10/03/2021
**15**	Clinical	Pericardium	Pericarditis	22	10/03/2021
**16**	Clinical	Heart Blood	Putative Sepsis	22	11/03/2021
**17**	Environmental	Meat Rinsate	Unknown	unknow	23/06/2021
**18**	Clinical	Spleen	Bacteremia	15	14/06/2021
**19**	Clinical	Spleen	Bacteremia	15	14/06/2021
**20**	Clinical	Unknown	Bacteremia	15	14/06/2021
**21**	Clinical	Unknown	Bacteremia	15	14/06/2021
**22**	Clinical	Unknown	Bacteremia	15	26/03/2021
**23**	Clinical	Unknown	Bacteremia	15	14/06/2021
**24**	Clinical	Unknown	Bacteremia	15	13/06/2021
**25**	Clinical	Unknown	Bacteremia	15	13/06/2021
**26**	Clinical	Unknown	Bacteremia	15	13/06/2021
**27**	Clinical	Unknown	Bacteremia	15	14/06/2021
**28**	Clinical	Unknown	Bacteremia	15	14/06/2021
**29**	Clinical	Unknown	Bacteremia	15	14/06/2021
**30**	Clinical	Unknown	Bacteremia	15	14/06/2021
**31**	Clinical	Unknown	Bacteremia	15	14/06/2021
**32**	Healthy	Caeca	Healthy	15	14/06/2021
**33**	Healthy	Caeca	Healthy	15	14/06/2021
**34**	Healthy	Caeca	Healthy	15	14/06/2021
**35**	Diseased	Pericardium	Pericarditis	Unknow	04/03/2021
**36**	Clinical	Heart Blood	Pericarditis	29	22/04/2021
**37**	Clinical	Fermoral Head	Fermoral Chondritis	29	27/03/2021
**38**	Clinical	Caeca	Healthy	364	22/07/2021
**39**	Clinical	Caeca	Healthy	364	22/07/2021
**40**	Clinical	Caeca	Healthy	364	22/07/2021
**41**	Clinical	Caeca	Healthy	364	22/07/2021
**42**	Clinical	Caeca	Healthy	364	22/07/2021
**43**	Environmental	Retail Chicken	Healthy	N/A	30/09/2021
**44**	Environmental	Breast	Healthy	N/A	28/06/2021
**45**	Environmental	Breast	Healthy	N/A	28/06/2021
**46**	Environmental	Breast	Healthy	N/A	11/10/2021
**47**	Environmental	Pelvis	Healthy	N/A	11/10/2021
**48**	Environmental	Wing	Healthy	N/A	11/10/2021
**49**	Environmental	Wing	Healthy	N/A	11/10/2021
**50**	Environmental	Wing	Healthy	N/A	12/10/2021
**51**	Environmental	Wing	Healthy	N/A	11/10/2021
**52**	Environmental	Wing	Healthy	N/A	11/10/2021
**53**	Environmental	Wing	Healthy	N/A	13/10/2021

**54**	Environmental	Wing	Healthy	N/A	11/10/2021
**55**	Commensal	Breast	Healthy	N/A	23/08/2022
**56**	Commensal	Breast	Healthy	N/A	15/06/2021
**57**	Commensal	Breast	Healthy	N/A	20/08/2022
**58**	Commensal	Breast	Healthy	N/A	21/08/2022
**59**	Commensal	Breast	Healthy	N/A	Unknown
**60**	Commensal	Breast	Healthy	N/A	Unknown
**61**	Commensal	Breast	Healthy	N/A	Unknown
**62**	Commensal	Wing	Healthy	N/A	15/03/2021
**63**	Commensal	Rinsate	Healthy	N/A	Unknown
**64**	Commensal	Rinsate	Healthy	N/A	21/02/2022
**65**	Commensal	Rinsate	Healthy	N/A	Unknown
**66**	Commensal	Rinsate	Healthy	N/A	22/02/2022
**67**	Clinical	Unknown	Lameness - undiagnosed	23	23/02/2022
**68**	Clinical	Unknown	Lameness - undiagnosed	23	23/02/2022
**69**	Clinical	Spleen	Lameness - undiagnosed	23	23/02/2022
**70**	Clinical	Spleen	Lameness - undiagnosed	23	23/02/2022
**71**	Clinical	Spleen	Lameness - undiagnosed	23	23/02/2022
**72**	Clinical	Heart	Lameness - undiagnosed	23	23/02/2022
**73**	Clinical	Heart	Lameness - undiagnosed	23	23/02/2022
**74**	Clinical	Heart	Lameness - undiagnosed	23	23/02/2022
**75**	Clinical	Unknown	Unknown	Unknow	Unknown
**76**	Clinical	Unknown	Unknown	Unknow	Unknown
**77**	Clinical	Unknown	Unknown	Unknow	Unknown
**78**	Clinical	Unknown	Unknown	Unknow	04/01/2021
**79**	Clinical	Unknown	Unknown	Unknow	Unknown
**80**	Clinical	Unknown	Unknown	Unknow	Unknown
**81**	Clinical	Unknown	Unknown	Unknow	05/01/2021
**82**	Clinical	Unknown	Unknown	Unknow	Unknown
**83**	Clinical	Unknown	Unknown	Unknow	Unknown
**84**	Clinical	Unknown	Unknown	Unknow	Unknown
**85**	Clinical	Unknown	Unknown	Unknow	Unknown
**86**	Clinical	Unknown	Unknown	Unknow	Unknown
**87**	Clinical	Unknown	Unknown	Unknow	Unknown
**88**	Clinical	Unknown	Unknown	Unknow	Unknown
**89**	Clinical	Unknown	Unknown	Unknow	Unknown
**90**	Clinical	Unknown	Unknown	Unknow	Unknown
**91**	Clinical	Unknown	Unknown	Unknow	Unknown
**92**	Environmental	Processing House	Unknown	Unknow	22/04/2022
**93**	Environmental	Processing House	Unknown	Unknow	22/04/2022
**94**	Environmental	Processing House	Unknown	Unknow	22/04/2022
**95**	Environmental	Processing House	Unknown	Unknow	23/04/2022
**96**	Environmental	Processing House	Unknown	Unknow	22/04/2022
**97**	Environmental	Processing House	Unknown	Unknow	22/04/2022
**98**	Environmental	Processing House	Unknown	Unknow	24/04/2022
**99**	Clinical	Unknown	Unknown	15 day	27/04/2022
**100**	Clinical	Unknown	Unknown	15 day	27/04/2022
**101**	Clinical	Unknown	Unknown	15 day	27/04/2022
**102**	Clinical	Unknown	Unknown	15 day	27/04/2022
**103**	Clinical	Unknown	Unknown	15 day	27/04/2022
**105**	Clinical	Unknown	Unknown	15 day	27/04/2022
**106**	Clinical	Unknown	Unknown	15 day	27/04/2022
**107**	Clinical	Unknown	Unknown	15 day	27/04/2022
**108**	Clinical	Unknown	Unknown	15 day	27/04/2022
**109**	Clinical	Unknown	Unknown	15 day	27/04/2022
**110**	Clinical	Unknown	Unknown	15 day	27/04/2022
**111**	Clinical	Unknown	Unknown	15 day	27/04/2022

**112**	Clinical	Unknown	Unknown	15 day	27/04/2022
**113**	Clinical	Unknown	Unknown	15 day	27/04/2022
**114**	Clinical	Unknown	Unknown	15 day	27/04/2022
**115**	Clinical	Unknown	Unknown	15 day	27/04/2022
**116**	Clinical	Unknown	Unknown	15 day	27/04/2022
**117**	Clinical	Unknown	Unknown	15 day	27/04/2022
**118**	Clinical	Unknown	Unknown	15 day	27/04/2022
**119**	Clinical	Unknown	Unknown	15 day	27/04/2022
**120**	Clinical	Unknown	Unknown	15 day	27/04/2022
**121**	Clinical	Unknown	Unknown	15 day	27/04/2022
**122**	Clinical	Unknown	Unknown	15 day	27/04/2022
**123**	Clinical	Unknown	Unknown	15 day	27/04/2022
**124**	Clinical	Unknown	Unknown	15 day	27/04/2022
**125**	Clinical	Unknown	Unknown	15 day	27/04/2022
**126**	Clinical	Unknown	Unknown	15 day	27/04/2022
**127**	Clinical	Unknown	Unknown	15 day	27/04/2022
**128**	Clinical	Unknown	Unknown	15 day	25/04/2022
**129**	Clinical	Unknown	Unknown	15 day	27/04/2022
**130**	Clinical	Unknown	Unknown	15 day	27/04/2022
**131**	Clinical	Unknown	Unknown	15 day	26/04/2022
**132**	Clinical	Unknown	Unknown	15 day	27/04/2022
**133**	Clinical	Unknown	Unknown	15 day	27/04/2022
**134**	Clinical	Unknown	Unknown	15 day	27/04/2022
**135**	Clinical	Unknown	Unknown	15 day	27/04/2022
**136**	Clinical	Unknown	Unknown	15 day	27/04/2022
**137**	Clinical	Unknown	Unknown	15 day	27/04/2022
**138**	Clinical	Unknown	Unknown	15 day	27/04/2022
**139**	Clinical	Unknown	Unknown	15 day	27/04/2022
**140**	Clinical	Unknown	Unknown	15 day	27/04/2022
**141**	Clinical	Unknown	Unknown	15 day	27/04/2022
**142**	Clinical	Unknown	Unknown	15 day	27/04/2022
**143**	Clinical	Unknown	Unknown	15 day	27/04/2022
**144**	Clinical	Unknown	Unknown	15 day	27/04/2022
**145**	Clinical	Unknown	Unknown	15 day	27/04/2022
**146**	Clinical	Unknown	Unknown	15 day	27/04/2022
**147**	Clinical	Unknown	Unknown	15 day	27/04/2022
**148**	Clinical	Unknown	Unknown	15 day	27/04/2022
**149**	Clinical	Unknown	Unknown	15 day	27/04/2022
**150**	Clinical	Unknown	Unknown	15 day	27/04/2022
**151**	Clinical	Unknown	Unknown	15 day	27/04/2022
**152**	Clinical	Unknown	Unknown	15 day	27/04/2022
**153**	Clinical	Unknown	Unknown	15 day	27/04/2022
**154**	Clinical	Unknown	Unknown	15 day	27/04/2022
**155**	Clinical	Unknown	Unknown	15 day	27/04/2022
**156**	Clinical	Unknown	Unknown	15 day	27/04/2022
**157**	Clinical	Unknown	Unknown	15 day	27/04/2022
**158**	Clinical	Unknown	Unknown	15 day	27/04/2022
**159**	Clinical	Unknown	Unknown	15 day	27/04/2022
**160**	Clinical	Unknown	Unknown	15 day	27/04/2022
**161**	Clinical	Unknown	Unknown	15 day	27/04/2022
**162**	Clinical	Unknown	Unknown	15 day	27/04/2022
**163**	Clinical	Unknown	Unknown	15 day	27/04/2022
**164**	Clinical	Unknown	Unknown	15 day	27/04/2022
**165**	Clinical	Unknown	Unknown	15 day	27/04/2022
**166**	Clinical	Unknown	Unknown	15 day	28/04/2022
**167**	Clinical	Unknown	Unknown	15 day	27/04/2022
**168**	Clinical	Unknown	Unknown	15 day	27/04/2022

**169**	Clinical	Unknown	Unknown	15 day	27/04/2022
**170**	Clinical	Unknown	Unknown	15 day	27/04/2022
**171**	Clinical	Unknown	Unknown	15 day	27/04/2022
**172**	Clinical	Unknown	Unknown	15 day	27/04/2022
**173**	Clinical	Unknown	Unknown	15 day	27/04/2022
**174**	Environmental	Caeca	Unknown	Unknow	23/06/2022
**175**	Clinical	Hock	Unknown	Unknow	27/05/2022
**176**	Clinical	Hock	Unknown	Unknow	27/05/2022
**177**	Clinical	Heart	Unknown	Unknow	27/05/2022
**178**	Clinical	Hock	Unknown	Unknow	27/05/2022
**179**	Clinical	Hock	Unknown	Unknow	27/05/2022
**180**	Clinical	Knee	Unknown	Unknow	27/05/2022
**181**	Clinical	Knee	Unknown	Unknow	27/05/2022
**182**	Clinical	Heart	Unknown	Unknow	27/05/2022
**183**	Clinical	Heart	Unknown	Unknow	27/05/2022
**184**	Clinical	Hock	Unknown	Unknow	27/05/2022
**185**	Clinical	Knee	Unknown	Unknow	27/05/2022
**186**	Clinical	Heart	Unknown	Unknow	27/05/2022
**187**	Clinical	Heart	Unknown	Unknow	27/05/2022
**188**	Clinical	Hock	Unknown	Unknow	27/05/2022
**189**	Clinical	Heart	Unknown	Unknow	27/05/2022
**190**	Clinical	Heart	Unknown	Unknow	28/05/2022

Table S3. List of antibiotics used in the study.

Product name	Provider	Code and/or CAS
Amoxicillin	Bioserv	A122-25G
Ampicillin sodium salt BP1760-25	Fisher bioreagents	69-52-3
Avilamycin	trc	TRC-A794655
Bacitracin	BioServ	BS-3759A/1405-87-4
Cefotaxime sodium salt C-7912	Sigma	64485-93-4
Chloramphenicol	Acros organics	227920250/56-75-7
Ciprofloxacin hydrochloride monohydrate	BioServ	BS-2367A
Daptomycin	trc	TRC-D193350/103060-53-3
Doxycycline hyclate	BioServ	BS-1726A/24390-14-5
Enrofloxacin	MP Biomedicals	93106-60-6
Erythromycin E6376	Sigma	102188727/114-07-8
Fosfomycin dissodium salt	trc	TRC-F72755
Gentamicin sulfate A1492	BioChemica	1405-41-0
Imipenem	BioServ	BS-1119A
Levofloxacin	BioServ	BS-6417A/100986-85-4
Lincomycin hydrochloride	BioServ	BS-3892A/859-18-7
Linezolid	Acros organics	460590010/165800-03-3
Norfloxacin	BioServ	BS-8833A
Nitrofurantoin	Bioserv	BS-0512A/67-20-9
Phenoxymethylpenicillinic acid potassium salt	fluorochem	132-98-9
Rifampicin R3501	Sigma	13292-46-1
Spectinomycin sulfate	BioServ	BS-0510A/23312-56-3
Streptomycin sulfate salt	BioServ	BS-7700A
Spiramycin	trc	TRC-S682373/8025-81-8
Sulfamethoxazole sodium salt	BioServ	BS-2831A/4563-84-2
Teicoplanin	BioServ	BS-8806A
Tetracycline hydrochloride	PhytoTech	T859/64-75-5
Tiamulin fumarate (Vetranal)	Merck	46959-100MG
Tigecycline	thermoscientific	220620-09-7
Trimethoprim	BioServ	BS-3656A/738-70-5
Tylosin tartrate	BioServ	BS-2429A
Vancomycin hydrochloride	Sigma	1404-93-9

Table S4. Occurrence of mannitol metabilism genes amongst *E. cecorum* UK isolates

Strain	Mannitol activator Blg family	PTS mannitol-specific IIA component	Mannitol-1-phosphate 5-dehydrogenase (EC 1.1.1.17)
AP_4	No	No	No
AP_6	No	No	No
AP_10	No	No	No
AP_12	No	No	No
AP_13	No	No	No
AP_17	No	No	No
AP_22	No	No	No
AP_37	No	No	No
AP_40	Yes	Yes	Yes
AP_43	Yes	Yes	Yes
AP_45	No	No	No
AP_48	No	No	No
AP_50	Yes	Yes	Yes
AP_53	Yes	Yes	Yes
AP_55	No	No	No
AP_56	No	No	No
AP_57	No	No	No
AP_58	No	No	No
AP_62	No	No	No
AP_64	No	No	No
AP_66	No	No	No
AP_67	No	No	No
AP_73	No	No	No
AP_78	No	No	No
AP_81	No	No	No
AP_92	No	No	No
AP_95	No	No	No
AP_98	No	No	No
AP_128	No	No	No
AP_131	No	No	No
AP_149	No	No	No
AP_166	No	No	No
AP_174	No	No	No
AP_182	No	No	No
AP_190	No	No	No
DSMZ109010	No	No	No
DSMZ100908	No	No	No
DSMZ11364	No	No	No
DSMZ20683	No	No	No

Table S5 Prophage gene content within UK *E. cecorum* isolates

Phage	Lysogeny			Replication	Morphogenesis			DNA packaging	Lysis
	Repressor & anti-repressor	Integrase	Excisionase		Portal	Head	Tail		
AP_22	√	√	X	√	√	√	√	√	√
AP_50	X	X	X	√	X	√	X	X	X
AP_56_P1	X	X	X	√	√	√	√	√	√
AP_56_P2	√	√	X	√	√	√	√	√	√
AP_57_P1	X	X	X	√	√	√	√	√	√
AP_57_P1	X	√	X	√	√	√	√	√	√
AP_64_P1	X	√	X	√	√	√	√	√	√
AP_64_P2	X	X	X	√	√	√	√	√	X
AP_64_P3	X	√	X	√	X	X	X	X	X
AP_66_P1	√	√	X	√	√	√	√	√	√
AP_66_P2	X	√	X	√	√	√	√	√	√
AP_66_P3	X	X	X	X	√	√	√	X	X
AP_67_P1	X	√	X	√	√	√	√	√	√
AP_73_P1	X	√	X	√	√	√	√	√	√
AP_92_P1	X	√	X	√	√	√	√	√	√
AP_92_P2	X	√	X	√	√	√	√	√	√
AP_92_P3	X	X	X	√	√	√	√	√	√
AP_98_P1	X	√	X	√	√	√	√	√	√
AP_98_P2	X	√	X	√	√	√	√	√	√
AP_98_P3	X	X	X	√	√	√	√	√	√
NCTC_12421	√	√	√	√	√	√	√	√	√
NCTC_12421	√	√	X	√	√	√	√	√	√

√	Present
X	Absent
